# Lifetime Duration of Exposure to Biomechanical Factors at Work as a Mediator of the Relationship Between Socioeconomic Position and Walking Speed

**DOI:** 10.3389/fpubh.2020.00412

**Published:** 2020-11-12

**Authors:** Angelo d'Errico, Fulvio Ricceri, Alexis Descatha, Annette Leclerc, Yves Roquelaure, Marcel Goldberg

**Affiliations:** ^1^Local Health Unit TO3, Epidemiology Department, Turin, Italy; ^2^Department of Clinical and Biological Sciences, University of Turin, Turin, Italy; ^3^Inserm, Population-Based Epidemiologic Cohorts Unit, UMS 011, Paris, France; ^4^University of Angers, Angers, France; ^5^Paris Descartes University, Paris, France

**Keywords:** walking speed, socioeconomic position, mediation analysis, ergonomics, work

## Abstract

The study aimed to assess the proportion mediated by the duration of exposure to ergonomic factors at work on the relationship between socioeconomic position (SEP) and low walking speed. This cross-sectional study was performed on data collected at baseline on 19,704 men and 20,273 women 45–70 years old, currently or previously employed, enrolled in the Constances cohort. SEP was assigned through current or last occupation, categorized in three classes, based on the European Socioeconomic Classification. Walking speed was assessed through one measurement of normal walking for 3 m and dichotomized at the lowest quintile of the sex- and age- (5-year) specific distribution. Self-reported workplace exposure throughout working life to repetitive work, intense physical work, and lifting/carrying heavy loads was used to assess the duration of exposure to each factor, categorized in four classes. Through Poisson regression models, adjusted for BMI, smoking, alcohol intake, hypertension, physical activity, diabetes, cardiovascular diseases, and a cognitive score, the attenuation in the prevalence ratio (PR) of low walking speed by SEP produced by the inclusion of duration of exposure to each factor was evaluated. The mediating effect of work ergonomic exposures on the relationship between SEP and low walking speed was assessed using the weighted method by Vanderweele. In the fully adjusted model without ergonomic exposures, both men and women in the middle and the lowest SEP had a significantly increased risk of low walking speed compared with those in the highest SEP (men: PR = 1.30 and PR = 1.46, respectively; women: PR = 1.24 and PR = 1.45, respectively). The inclusion in separate regression models of exposure duration to repetitive work, intense physical work, and handling of heavy loads produced modest risk attenuations in both men and women, all smaller or around 10%. Mediation analysis revealed in both sexes significant mediation effects for most ergonomic exposures considered, although also with low mediation effects. Significant differences in walking speed by SEP were observed in this large sample, but the proportion of such differences explained by the duration of exposure to ergonomic factors at work was low using either the risk attenuation or the mediation analysis methods.

## Introduction

Physical functioning is an important asset of older subjects, which influences their autonomy and their performance in daily life, such as participation in social activities and independent living (1).

Several studies have observed differences in physical functioning between socioeconomic groups among older subjects, with lower functioning among more disadvantaged people (2–4).

Walking speed has often been used as a marker of physical performance because it is an objective indicator assessed through a simple test, which has been shown to predict disability, dementia, and mortality in older populations (5–8), as well as incidence of cardiovascular disorders (9, 10). Furthermore, gait speed has been reported to be correlated with psychological well-being (11), self-rated health (12), and cognitive capacity (13, 14).

Low walking speed has been associated consistently with low socioeconomic position (SEP) in epidemiological studies (15–18), especially with adult SEP (19).

Besides low SEP, several risk factors have been reported to increase the risk of reduced walking speed, including behavioral risk factors, such as smoking, physical inactivity, obesity (20), other cardiovascular risk factors (21), hypertension and cerebral small vessels disease (22), high C-reactive protein levels (23), genetic factors (24), and osteoarticular diseases, especially knee osteoarthritis (25).

In a recent study from the LIFEPATH project, it was found that 60-year-old subjects with the lowest SEP had the same walking speed as subjects with the highest SEP, 6.6 years older among men and 4.6 years older among women (20). These results were derived from an analysis adjusted for a set of risk factors indicated by the World Health Organization (WHO) as targets to reduce premature mortality from chronic diseases by 25% in 2025, denominated “25 × 25” risk factors (high alcohol consumption, insufficient physical activity, tobacco use, high blood pressure, high salt intake, diabetes, and obesity) (26). Therefore, other factors may be implicated in determining the lower walking speed observed among older subjects with more disadvantaged SEP.

Exposure to some occupational ergonomic factors also seems to reduce walking speed at an older age. Previous heavy manual work was associated with lower gait speed in one study (27), whereas in another study, manual longest-held occupation during lifetime was associated with reduced performance using the Short Physical Performance Battery test, which includes walking speed, together with the chair stand test and the balance test (28). Two other studies found opposite effects of long-term occupational and leisure-time physical activity (LTPA) during midlife on self-reported mobility during old age, with the former associated with an increased risk and the latter with a decreased risk of poor physical functioning (29, 30). It has been hypothesized that the health effects of heavy physical work may differ from those of LTPA (known as the “physical activity paradox”), as the former often involves static postures and heavy lifting for long periods, and is performed with insufficient recovery time and poor control of the workers on tasks, speed, and schedule (31).

Given that manual work and, in general, exposure to biomechanical factors in the workplace is more prevalent among workers in more socially disadvantaged positions, it is of interest to assess the possible role of work-related factors as mediators of the relationship between SEP and walking speed. To our knowledge, only Plouvier et al. (32) assessed the mediating effect of ergonomic factors at work on the SEP–walking speed relationship, in a pilot cross-sectional study using baseline data from the Constances cohort; the authors, based on the risk attenuation obtained from the adjustment for ergonomic exposures, found that 40% of the differences in walking speed between extreme SEP categories among men were explained by exposure to carrying heavy loads, whereas no mediation effect was present among women for carrying heavy loads, nor in either sex for repetitive movements. However, this study included a relatively small number of subjects (n. 750) and did not control the analysis for behavioral factors, which may have led to an overestimation of SEP differences in walking speed explained by workplace exposures.

The objective of the present study was to assess the mediating role of ergonomic exposures on the relationship between SEP and walking speed on the much larger population enrolled in the Constances study up to the end of 2017, after taking into account WHO 25 × 25 risk factors (20) and health conditions. Based on the results in the literature on SEP differences in walking speed and in exposure to ergonomic factors at work, as well as on the findings of the study by Plouvier et al. (32), we hypothesize that exposure to ergonomic factors at work during a lifetime, in particular carrying/lifting heavy loads, would mediate a large proportion of SEP differences in walking speed, especially among men.

A secondary objective was to compare the mediation effect of biomechanical exposures at work estimated through mediation analysis with that computed through attenuation of the relative risk associated with SEP, as the risk attenuation method can give biased results in case of interaction effects between the exposure and the mediator (33).

## Materials and Methods

### Data Collection

#### Study Population

Data on men and women enrolled in the Constances study were collected through a questionnaire on lifestyle, health, physical limitations, social and personal characteristics, and lifetime job history (34, 35). These subjects also underwent a clinical examination in one of the 19 participating health screening centers, and those older than 44 years were also submitted to additional tests on physical and cognitive functions. The global participation rate in Constances was 7.3%, which is usual in studies in which participants are asked to travel to a medical center. As we had access to data from French administrative, social, and health databases, we were able to compare participants and non-participants. Participation was higher among women and people with a higher level of education. It was lower among people suffering from several diseases, such as diabetes, cardiovascular disease, HIV infection, or psychiatric disorders (36).

The study population was composed of all subjects 45–70 years old, currently or formerly employed (mean: 57.6 ± 7.2 years), who are enrolled in the Constances cohort, who were assessed for walking speed at baseline and had complete information on the present occupation, if still working, or on the last occupation performed, if not working, and on ergonomic exposures during a lifetime, WHO 25 × 25 risk factors, cognitive function, height, and health conditions (19,704 men and 20,273 women).

#### Walking Speed Measurement

Walking speed was measured, during the health examination, in meters/second across 3 m using a photocell, with a 1-m zone to accelerate and decelerate before and after the stop line, asking subjects to walk at their normal speed and allowing for one trial before measurement.

#### Socioeconomic Position

SEP was assigned to the study population using the current, or last occupation if not employed, which was self-reported by the subjects. Occupations were categorized in three classes, based on the European Socioeconomic Classification (ESeC), that were “Higher occupations,” “Intermediate occupations,” and “Routine and manual occupations,” following the occupational class categorization used in the LIFEPATH project (37).

Briefly, “Higher occupations” included subjects belonging to ESeC class 1 (large employers, higher professionals and managers), ESeC class 2 (lower professionals and managers, and higher-grade technical and supervisory occupations), and ESeC class 3 (higher-grade clerical, services, and sales workers). In “Intermediate occupations” were classified workers in ESeC class 4 (small employers and self-employed outside of agriculture), ESeC class 5 (farmers, self-employed in agriculture), and ESeC class 6 (lower-supervisory and technical occupations). Last, the class of “Routine and manual occupations” included workers belonging to ESeC class 7 (lower-clerical, services, and sales workers), ESeC class 8 (skilled workers), and ESeC class 9 (semi- and unskilled workers).

#### Occupational Biomechanical Exposures

Ergonomic exposures considered in the study were those for which self-reported information on lifetime exposure was collected at baseline, that is, repetitive work, intense physical work, and carrying or handling heavy loads, together with the year of start and end of each exposure. Based on this information, years of exposure to each factor were summed across all working periods to build a cumulative duration of exposure for each factor, which was categorized in four classes (0, 0.1–10, 10.1–20, and 20.1 + years).

#### World Health Organization 25 × 25 Risk Factors

Among lifestyle factors, the risk factors listed in the WHO 25 × 25 strategy were selected as potential confounders, to conduct analyses comparable with those by Stringhini et al. (20). In detail, the following behavioral factors and conditions were considered in the study:

- Alcohol consumption: self-reported, measured in alcohol units/week; participants were categorized as abstainers (0 unit/week), moderate (1–21 units/week for men, 1–14 for women), and heavy (> 21 units/week for men, >14 for women) drinkers;
- LTPA: self-reported sports activities (never, more than half an hour per week);
- Body mass index (BMI): based on weight and height measurement, categorizing normal BMI as BMI < 25, overweight as 25 ≤ BMI < 30, and obesity as BMI ≥ 30; for this analysis, underweight subjects, defined as those with a BMI < 18.5, were aggregated to subjects with normal BMI because of their small number [32 among males [0.2%] and 285 among females [1.4%]];
- Smoking habit: self-reported and categorized as never, current, and former;
- Hypertension: the presence of at least one of the following conditions: systolic blood pressure above 140 mmHg, diastolic blood pressure above 90 mmHg, or self-reported current intake of antihypertensive medication;
- Diabetes: fasting blood glucose levels above 7 mmol/L or self-reported use of antidiabetic drugs.

Salt intake was not considered, as it was unavailable in the Constances data.

#### Health Conditions

The presence of cardiovascular diseases (including arteritis, coronary heart disease, and stroke) was assessed through self-report at the baseline health examination.

#### Cognitive Score

A cognitive score was computed for each subject above 44 years, based on measurements conducted by neuropsychologists through multiple tests (34).

### Statistical Analysis

For descriptive analyses, trends by SEP of the categorical duration of exposure to ergonomic factors and walking speed were evaluated through a non-parametric test for trend, developed from the Wilcoxon rank-sum test, computed as the sum of ranks for each group weighted by its ordered position (38).

The lowest quintile of the sex-specific distribution of walking speed was established as the cut-off, distinguishing low and normal speed, considering those in the lowest 20% of the distribution as affected by low walking speed (below 1.09 m/s among men and below 1.05 m/s among women).

Data were analyzed using Poisson robust regression models with SEP as the independent variable and the dichotomized measure of walking speed as the dependent variable.

Two types of analysis were conducted to assess the mediator effect of exposure to each occupational factor, one based on the risk attenuation produced by the inclusion of the factor in the Poisson robust regression model and the other one through formal mediation analysis.

Regarding Poisson robust regression, a first model was adjusted for age, height, smoking status, BMI, diabetes, LTPA, hypertension, alcohol intake, presence of cardiovascular diseases, and the cognitive score; in a second model, also information on the categorical duration of exposure to the three biomechanical factors at work was included in the models (never, up to 10 years, 10.1–20 years, and more than 20 years), to assess the attenuation of the prevalence ratio (PR) of low walking speed by SEP produced by adjustment for these work factors. It was not possible to introduce duration of exposure to all the three ergonomic factors together in one model, as intense physical work and carrying/handling heavy load were too strongly correlated (*r* = 0.72).

**Figure 1:**
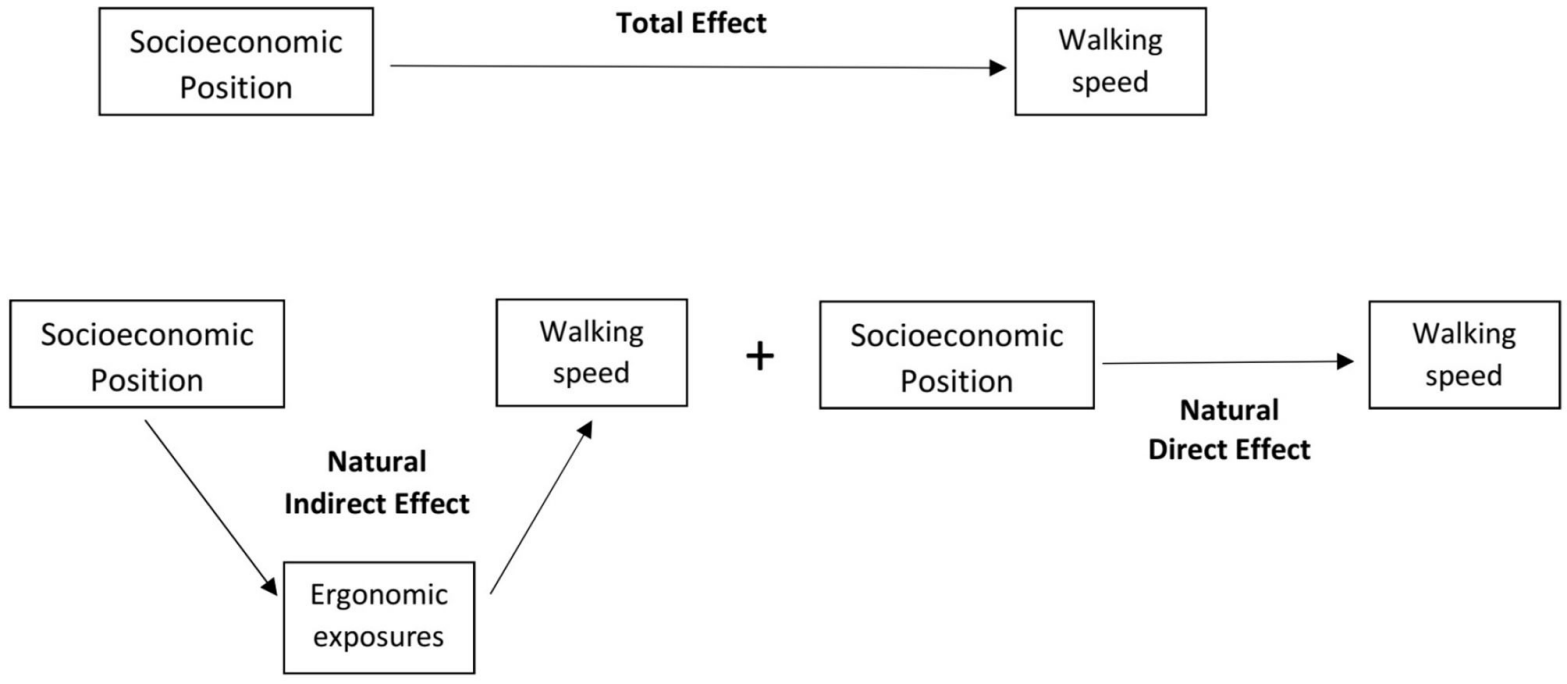
Components of the mediation analysis. Total Effect of socioeconomic position (SEP) on walking speed, decomposed in Natural Direct Effect and Natural Indirect Effect of SEP, the latter mediated by exposure to ergonomic factors at work.

The mediation analyses were performed using the counterfactual approach (39). Briefly, given an exposure A, a mediator M, and an outcome Y, each subject has two counterfactual events: Y1, if the subject has been exposed to A, and Y0 if not (supposing, for simplicity, binary exposures, mediators, and outcomes). Similarly, M1 and M0 are the counterfactuals of the mediator, in the presence or absence of the exposure, respectively, and Yam is the counterfactual event for the scenario A = a and M = m. The Total Effect (TE) is the difference between the two means of the counterfactual events, Y1M1-Y0M0, and it is estimated by E(Y1M1-Y0M0). The Natural Direct Effect (NDE) is the contrast between the counterfactual outcome if the individual were exposed to A and the counterfactual outcome if the same individual were not exposed to A, with the mediator assuming whatever value it would have taken at the reference value of absence of the exposure. Setting the exposure as present, the Natural Indirect Effect (NIE) is defined as the contrast between the counterfactual outcome if the mediator assumed the value it would have taken in the presence of the exposure and the counterfactual outcome if the mediator assumed the value it would have taken in the absence of the exposure. In other words, the NIE compares what would have happened to the outcome, having set the exposure as present, if the mediator took the value observed when the exposure is present with what would have happened if the mediator took the value when the exposure is absent. In our example, the TE represents the total effect of occupation on walking speed, which is decomposed in a direct effect (NDE), and in an indirect effect (NIE), which is the effect of the mediators (Figure 1).

Bias-corrected 95% confidence intervals have been computed for the mediation analysis (40), as they are suggested as best confidence intervals in a bootstrap procedure (41).

To evaluate whether the mediation effect of the ergonomic factors was affected by adjustment for 25 × 25 risk factors and other covariates, which could have resulted in an overadjustment, a sensitivity analysis was also performed to estimate the risk attenuation of the PRs by SEP produced by the inclusion of each ergonomic factor in a model adjusted only for age and health center, as in the article by Plouvier et al. (32).

## Results

### Descriptive Statistics

The frequency distribution of the characteristics of the study population by sex revealed among men a higher proportion of subjects belonging to higher SEP, of current smokers, and of overweight people, or affected by hypertension, diabetes, or cardiovascular diseases, compared with women, whereas the distribution of the other covariates was quite comparable across sex (Table 1).

**Table 1:** Prevalence of characteristics of the study population, by sex.

**Covariates**	**Men**	**Women**
	**N (%)**	**N (%)**
**Socioeconomic position**		
High	8,468 (43.0)	5,193 (25.6)
Middle	6,589 (33.4)	7,775 (38.4)
Low	4,647 (23.6)	7,305 (36.0)
**Employment status**		
Employed	11,279 (57.2)	11,727 (57.8)
Not employed	8,425 (42.3)	8,546 (42.2)
**Alcohol consumption**		
Abstainer	1,886 (9.6)	3,540 (17.5)
Moderate	12,469 (63.3)	11,858 (58.5)
Heavy	5,349 (27.2)	4,875 (24.1)
**BMI**		
<18.5	32 (0.2)	285 (1.4)
18.5–24.9	7,711 (39.1)	12,127 (59.8)
25–29.5	9,045 (45.9)	5,421 (26.7)
30+	2,916 (14.8)	2,440 (12.0)
**Hypertension**		
No	11,522 (58.5)	15,344 (75.7)
Yes	8,182 (41.5)	4,929 (24.3)
**Diabetes**		
No	18,578 (94.3)	19,899 (98.2)
Yes	1,126 (5.7)	374 (1.8)
**Leisure physical activity**		
No	4,467 (22.7)	3,845 (19.0)
Yes	15,237 (77.3)	16,428 (81.0)
**Smoking habit**		
Never	7,424 (37.7)	10,073 (49.7)
Former	2,524 (12.8)	2,675 (13.2)
Current	9,756 (49.5)	7,525 (37.1)
**Cardiovascular diseases**		
No	18,703 (94.9)	19,997 (98.6)
Yes	1,001 (5.1)	276 (1.4)
	**Mean (sd)**	**Mean (sd)**
**Age**	57.6 (7.1)	57.1 (7.0)
**Height (cm)**	174.9 (6.5)	162.1 (6.2)
**Cognitive score**	0.04 (0.59)	0.05 (0.56)

*Men and women 45–70 years (19,704 men and 20,273 women) employed or previously employed*.

For all the ergonomic factors examined, there were strong differences by SEP for ever exposure (exposure for at least 1 year) and categorical duration of exposure in both men and women (Table 2), with significant trends of increasing prevalence and duration of exposure with decreasing SEP (test for trend: *p* < 0.001 for ever exposure and categorical exposure duration for all factors in both sexes) (Table 2).

**Table 2:** Proportion of workers exposed to biomechanical factors at work and walking speed by socioeconomic position (SEP) and sex.

	**Men**			**Women**		
**Exposures**	**High SEP N (%)**	**Middle SEP N (%)**	**Low SEP N (%)**	**High SEP N (%)**	**Middle SEP N (%)**	**Low SEP N (%)**
**Repetitive movements^a^**						
Never	8,321 (98.3)	6,262 (95.0)	3,893 (83.8)	5,154 (99.2)	7,604 (97.8)	6,419 (87.9)
1–10 years	103 (1.2)	221 (3.4)	327 (7.0)	24 (0.5)	111 (1.4)	468 (6.4)
10.1–20 years	20 (0.2)	62 (0.9)	165 (3.6)	10 (0.2)	39 (0.5)	191 (2.6)
>20 years	24 (0.3)	24 (0.3)	262 (5.6)	5 (0.1)	21 (0.3)	227 (3.1)
**Intense physical work**^a^						
Never	7,832 (92.5)	5,052 (76.7)	2,416 (52.0)	4,972 (95.7)	6,781 (87.2)	5,867 (81.7)
1–10 years	355 (4.2)	563 (8.5)	512 (11.0)	97 (1.9)	265 (3.4)	513 (7.0)
10.1–20 years	137 (1.6)	369 (5.6)	503 (10.8)	38 (0.7)	217 (2.8)	345 (4.7)
>20 years	144 (1.7)	605 (9.2)	1216 (26.2)	86 (1.7)	512 (6.6)	480 (6.6)
**Lifting/carrying heavy loads**^a^						
Never	7,781 (91.9)	4,772 (72.4)	2,078 (44.7)	4,920 (94.7)	6,613 (85.1)	5,767 (79.0)
0.1–10 years	387 (4.6)	629 (9.6)	612 (13.2)	132 (2.5)	343 (4.4)	610 (8.4)
10.1–20 years	149 (1.8)	465 (7.1)	566 (12.2)	57 (1.1)	265 (3.4)	426 (5.8)
>20 years	151 (1.8)	723 (11.0)	1391 (29.9)	84 (1.6)	554 (7.1)	502 (6.9)
	**Mean (sd)**	**Mean (sd)**	**Mean (sd)**	**Mean (sd)**	**Mean (sd)**	**Mean (sd)**
Walking speed—Normal (m/sec)^a^	1.31 (0.50)	1.27 (0.42)	1.25 (0.47)	1.26 (0.29)	1.24 (0.32)	1.23 (0.52)

a *p-value for trend: < 0.001*.

*Men and women 45–70 years employed or previously employed*.

Among men exposed to the ergonomic factors considered, average (st. dev.) cumulative durations of exposure were 13.2 (11.8) years for repetitive work, 19.4 (12.6) years for intense physical work, and 19.5 (12.6) years for carrying/lifting heavy loads, whereas corresponding figures among women were 12.6 (11.1) years for repetitive work, 18.2 (11.9) years for intense physical work, and 17.2 (11.4) years for carrying/lifting heavy loads.

Mean (st. dev.) time values elapsed since last exposure were, among men, 18.1 (14.9) years for repetitive work, 14.9 (13.8) years for intense physical work, and 14.4 (13.5) years for carrying/lifting heavy loads; among women, years since last exposure were 19.4 (15.0) for repetitive work, 11.0 (12.1) for intense physical work, and 11.1 (12.1) for carrying/lifting heavy loads.

Mean walking speed was higher in higher SEP strata, with a significant trend with increasing SEP in both sexes (test for trend: *p* < 0.001 for both sexes), although differences were quite small (Table 2).

### Association Between Exposure to Biomechanical Factors at Work and Low Walking Speed

#### Men

Exposure to repetitive work was associated with low walking speed but only for the categories of exposure duration up to 20 years (0.1–10 years: PR = 1.30; 10.1–20 years: PR = 1.61), whereas the PR for the longest one was close to one (PR = 1.06) (Table 3). Intense physical work increased the risk of low walking speed in the categories of 10–20 and > 20 years duration significantly, with similar risks (PR = 1.27 and PR = 1.23) but not for the duration up to 10 years (PR = 1.09).

**Table 3:** Prevalence ratios (PR) of low walking speed by categorical duration of exposure to ergonomic factors and sex.

	**Repetitive work**		**Intense physical work**		**Lifting/Carrying heavy loads**	
**Exposure**	**PR^a^^,^^b^**	**95% CI**	**PR^a^^,^^b^**	**95% CI**	**PR^a^^,^^b^**	**95% CI**
**Men– (ref.: never exposed)**	**1**	**–**	**1**	**–**	**1**	**–**
Exposed <= 10 years	1.30	1.15–1.48	1.09	0.98–1.22	1.19	1.08–1.31
Exposed 10.1–20 years	1.61	1.35–1.92	1.27	1.13–1.42	1.18	1.05–1.31
Exposed > 20 years	1.06	0.88–1.29	1.23	1.13–1.33	1.18	1.09–1.27
**Women (ref.: never exposed)**	**1**	–	**1**	–	**1**	–
Exposed <= 10 years	1.32	1.16–1.50	1.24	1.10–1.40	1.16	1.04–1.30
Exposed 10.1–20 years	1.24	1.01–1.53	1.25	1.08–1.44	1.07	0.93–1.23
Exposed > 20 years	1.58	1.34–1.86	1.18	1.06–1.31	1.15	1.04–1.28

a *Adjusted for age, height, smoking status, BMI, diabetes, leisure-time physical activity, hypertension, alcohol intake, cardiovascular diseases, and cognitive function score*.

b *Pearson's goodness of fit test: p = 1.00 for all models*.

Poisson regression models. Men and women 45–70 years employed or previously employed.

Carrying/lifting heavy loads increased the risk of low walking speed in all duration categories significantly, with excess risks all approaching 20%.

A significant trend across ordered categories of exposure duration was estimated for all ergonomic factors (*p* < 0.01).

#### Women

Among women, repetitive work increased the risk of low walking speed in all categories of duration significantly, with the highest PR observed for the longest duration (PR = 1.58) (Table 3).

Intense physical work was also associated with low walking speed in all exposure categories, with similar excess risks (around 20–25% increase) and no apparent trend in risk.

For carrying/lifting heavy loads, the risk was significantly increased for the lowest and the highest categories of exposure duration (PR = 1.16 and 1.15, respectively), whereas it was lower and not significant in the 10–20 years category (PR = 1.07).

Also for women, for all ergonomic factors, significant trends in risk across increasing categories of exposure duration were estimated (*p* < 0.01).

### Proportion of Low Walking Speed by Socioeconomic Position Mediated by Exposure to Biomechanical Factors at Work

#### Men

Among men, in the analysis adjusted for age, height, 25 × 25 risk factors, cognitive function score, and cardiovascular diseases, the PRs of low walking speed were 1.30 (95% CI: 1.21–1.39) for the middle occupational class and 1.46 (95% CI: 1.36–1.57) for the lowest class, compared with subjects with high SEP (Table 4).

**Table 4:** Prevalence ratios (PR) of low walking speed by socioeconomic position (SEP) and sex, unadjusted and adjusted for categorical duration of exposure to ergonomic factors at work.

	**Middle SEP**			**Low SEP**		
**Models**	**PR^b^**	**95% CI**	**% Risk attenuation**	**PR^b^**	**95% CI**	**% risk attenuation**
**MEN**						
Model 1^a^	1.30	1.21–1.39	REF.	1.46	1.36–1.57	REF.
Model 1 + repetitive work duration	1.28	1.20–1.37	6.7	1.42	1.32–1.53	8.7
Model 1 + physical effort duration	1.28	1.19–1.37	6.7	1.41	1.30–1.52	10.9
Model 1 + lifting/carrying heavy loads duration	1.28	1.20–1.37	6.7	1.43	1.32–1.55	6.5
**WOMEN**						
Model 1^a^	1.24	1.14–1.34	REF.	1.45	1.34–1.57	REF.
Model 1 + repetitive work duration	1.24	1.14–1.34	0.0	1.41	1.30–1.53	8.9
Model 1 + physical effort duration	1.22	1.13–1.33	8.3	1.42	1.32–1.54	6.7
Model 1 + lifting/carrying heavy loads duration	1.23	1.14–1.33	4.2	1.44	1.33–1.56	2.2

a *Model 1: adjusted for age, height, smoking status, BMI, diabetes, leisure-time physical activity, hypertension, alcohol intake, cardiovascular diseases, and cognitive function score*.

b *Pearson's goodness of fit test: p = 1.00 for all models*.

*Poisson regression models. Men and women 45–70 years employed or previously employed*.

Including ergonomic exposures in the regression model changed little the PRs for subjects in the middle SEP category, with each exposure producing the same small risk attenuation (6.7%), characterized by wide confidence intervals, overlapping with those of the reference regression model. Small risk attenuations were also observed for the lowest SEP category for each ergonomic exposure examined, with stronger risk attenuation found for intense physical effort (10.9%), and smaller for repetitive movements and lifting/carrying heavy loads (8.7 and 6.5%, respectively), but also in this case risk estimates had great uncertainty, with wide confidence intervals.

The results obtained from the mediation analysis (Table 5) showed a significant mediating effect only for physical effort for the middle SEP category (NIE 1.02, 95% CI: 1.01–1.03), whereas for the lowest SEP, a significant mediating effect was observed for exposure to intense physical effort (NIE 1.05, 95% CI: 1.02–1.07) and lifting/carrying heavy loads (NIE 1.03, 95% CI: 1.01–1.05), whereas for repetitive movements, it was only borderline significant (NIE 1.02, 95% CI: 1.00–1.03).

**Table 5:** Natural Direct (NDE), Natural Indirect (NIE), and Total Effect (TE) of categorical duration of exposure to ergonomic factors at work on the association between socioeconomic position (SEP) and low walking speed.

	**Men**				**Women**			
	**Middle SEP**		**Low SEP**		**Middle SEP**		**Low SEP**	
**MODELS**								
**Repetitive work**	**PR^a^**	**95% CI**	**PR^a^**	**95% CI**	**PR^a^**	**95% CI**	**PR^a^**	**95% CI**
Natural direct effect (NDE)	1.30	1.23–1.37	1.43	1.36–1.52	1.23	1.15–1.30	1.41	1.33–1.50
Natural indirect effect (NIE)	1.01	1.00–1.01	1.02	1.00–1.03	1.00	1.00–1.01	1.03	1.01–1.04
Total effect (TE)	1.31	1.24–1.37	1.46	1.37–1.54	1.23	1.16–1.32	1.45	1.36–1.55
**Intense physical effort**	**PR^a^**	**95% CI**	**PR^a^**	**95% CI**	**PR^a^**	**95% CI**	**PR^a^**	**95% CI**
Natural direct effect (NDE)	1.28	1.22–1.35	1.39	1.31–1.48	1.21	1.13–1.29	1.42	1.34–1.51
Natural indirect effect (NIE)	1.02	1.01–1.03	1.05	1.02–1.07	1.02	1.01–1.03	1.03	1.01–1.04
Total effect (TE)	1.31	1.24–1.48	1.46	1.37–1.54	1.23	1.16–1.32	1.45	1.37–1.55
**Lifting/Carrying heavy loads**	**PR^a^**	**95% CI**	**PR^a^**	**95% CI**	**PR^a^**	**95% CI**	**PR^a^**	**95% CI**
Natural direct effect (NDE)	1.29	1.22–1.35	1.41	1.33–1.50	1.22	1.14–1.30	1.43	1.34–1.52
Natural indirect effect (NIE)	1.01	1.00–1.03	1.03	1.01–1.06	1.01	1.00–1.02	1.02	1.00–1.03
Total effect (TE)	1.31	1.24–1.37	1.46	1.37–1.54	1.23	1.16–1.32	1.45	1.36–1.55

a *Prevalence ratio*.

*Men and women 45–70 years employed or previously employed*.

#### Women

Among women, PRs of 1.24 (95% CI: 1.14–1.34) for the middle SEP and of 1.45 (1.34–1.57) for the low SEP category were estimated from the reference Poisson regression model (Table 4).

For the middle SEP category, as for men, modest risk attenuations were found adjusting for physical effort (8.3%) or lifting/carrying heavy loads (4.2%) but not for repetitive work, whereas in the lowest SEP category, the risk attenuation was strongest for inclusion in the model of exposure to repetitive movements (8.9%), whereas smaller for intense physical effort (6.7%) and lifting/carrying heavy loads (2.2%). However, also among women, the attenuated PRs had wide confidence intervals overlapping with those of the reference model.

The mediation analysis revealed among women a significant indirect effect for exposure to physical effort for both SEP categories (middle SEP: NIE = 1.02, 95% CI: 1.01–1.03; low SEP: NIE = 1.03, 95% CI: 1.01–1.04) and repetitive work for the low SEP category (NIE = 1.03, 95% CI: 1.01–1.04), whereas the other results were only of borderline significance.

### Sensitivity Analysis

In both sexes, similar risk attenuations were computed adjusting for the ergonomic factors in a regression model including only age and health center, compared with those observed in the fully adjusted models (data not shown). As for the main analysis, the inclusion in the models of the categorical duration of exposure to each factor produced, in both men and women, risk attenuations of the PRs associated with middle or low SEP below or around 10%.

## Discussion

In a population of subjects in early old age, we found a significant SEP gradient in walking speed in both sexes, with an increased risk of ~45% for subjects in the lowest SEP, compared with the highest. This finding is consistent with the results of several studies, although age differences in study populations, SEP definition, and outcome measurement limit the direct comparability of SEP differences in gait speed found in the present study with those reported by other authors (15–17, 20, 32, 42).

Exposure to biomechanical factors at work was also associated with low walking speed, with significant trends in risks across ordered categories of exposure duration for all the three factors examined in both men and women. In several studies, walking speed was found inversely associated with exposure to workplace ergonomic factors or intense physical work, in contrast with LPTA, which instead has been reported to have a protective effect (27, 30, 32, 43), partly due to increased muscle strength (44). The opposite direction of the association with ergonomic factors at work, compared with LTPA, may be due, as discussed in the *Introduction*, to differences in type, frequency, and duration of the physical activity performed, which in the case of occupational physical activity is characterized mainly by static effort and constrained postures for long periods without sufficient pauses and recovery (30), features which may overload joint, tendon, and muscle structures, increasing the risk of developing musculoskeletal disorders (45, 46); these, in turn, would increase the likelihood of functional disability (47). Also, different studies have provided evidence of the possible detrimental effect of high occupational physical demand at work on cardiovascular biological endpoints, such as increased blood pressure (48), reduction in aerobic capacity (49, 50), and greater carotid atherosclerosis progression (51), all bodily changes that could also have an impact on functional mobility.

The proportion of SEP differences in gait speed mediated by exposure to workplace biomechanical factors during working life was low in the present study, with no more than 10% of the difference between the lowest and the highest SEP categories mediated by exposure to each factor.

The mediation analysis did not show substantial differences in the SEP proportion mediated by ergonomic factors compared with that computed through risk attenuation but allowed to examine whether such mediated fractions were statistically significant or not.

Similarly to our findings, in the previous study on walking speed conducted on Constances data, cited in the *Introduction*, no mediation effect was present in either sex for repetitive movements, nor among women for carrying heavy loads (32). Nonetheless, the mediated proportion computed in our study for lifting/carrying heavy loads among men seems at odds with the finding of Plouvier et al. (32) that 40% of the differences in walking speed between lowest and highest SEP among men was explained by exposure to carrying heavy loads. However, in their study, the attenuated risk estimate after accounting for carrying heavy loads was characterized by wide 95% confidence intervals, largely overlapping with those of the unadjusted risk estimate, leaving uncertainty whether the mediation effect of this ergonomic exposure was statistically significant or not. Also, the risk attenuation was assessed before adjusting the analysis for potential confounders, such as behavioral and cardiovascular risk factors, which may determine a possible overestimation of the mediation effect, in case the mediator is correlated with these confounders. On the other hand, the sensitivity analysis assessing the mediating effect of exposure to the same ergonomic factors through models adjusted only for age and health center, as in the study by Plouvier et al. (32), showed results similar to those of the main analysis.

Among other studies investigating this issue, Adamson et al. (52) found that the risk of locomotor disability in early old age by socioeconomic circumstances, measured through different SEP indicators (occupational class, car ownership, housing tenure, household income, and area deprivation), decreased by 36% among men and 22% among women, after adjusting for lifetime exposure to various occupational hazards, including ergonomic ones, such as bending and intense physical work, but also noise and microclimatic working conditions. However, the cross-sectional design of this study, together with the fact that both exposure to occupational hazards and locomotor disability were self-reported, leaves space to the possibility that the mediating effect of occupational exposures has been overestimated.

In another cross-sectional study, lifetime exposure to heavy physical work and kneeling/squatting explained only a rather small proportion (about 15% among women and < 10% among men) of the educational difference in mobility limitations assessed through test-based measures (53). The much higher proportion explained by self-reported measures in this study, compared with test-based measures, also suggests that in studies using self-reported measures of both physical functioning and exposure to occupational factors, the mediating effect of the latter may have been overestimated.

### Strengths

The present study was the first one, to our knowledge, that assessed the mediating effect of exposure to biomechanical factors at work on the relation between SEP and walking speed through formal mediation analysis. This feature is relevant, as in other studies the mediation effect of work exposures was estimated through risk attenuation, that is, evaluating the reduction in the relative risk by SEP adjusting for the potential mediator, a methodology whose results are often difficult to interpret, because of uncertainty on their statistical significance, and that can give biased results in case of interaction effects between the exposure and the mediator.

Another strength is the large sample used, which allowed assessing the mediating effect of occupational exposures stratifying by sex and controlling the analyses for many covariates, which are potential confounders of the associations investigated. Furthermore, the study population is a representative sample of the French population, which allows generalizing our findings to the whole population of corresponding age in this country who participated in paid work during their lifetime.

### Limitations

Among limitations, different studies have shown that self-reported exposure to ergonomic factors in the workplace has only moderate reproducibility and validity for most physical exposures (54–56), which is expected to decrease further for exposures occurred in a distant past, with the consequence of a non-differential misclassification of the exposure and an underestimation of the association with health outcomes. This potential bias has likely reduced the association between exposure to ergonomic factors and low walking speed in our study, with the consequences of an underestimation of the proportion of the excess risk mediated by these factors. Although differential misclassification of the duration of exposure to ergonomic factors may have occurred, it seems unlikely that subjects with low walking speed could have overestimated it, as the duration of exposure was based on self-reported information on the exact year of starting and end of each exposed period of work, and low walking speed was objectively measured.

Lack of information on the intensity of exposure to ergonomic factors, but only on duration, could have also led to non-differential misclassification of the actual cumulative dose of exposure to such factors. This bias would have also caused an underestimation of the true association between exposure to ergonomic factors and walking speed and, consequently, of their mediating effect, which would increase as the difference between exposure duration and cumulative dose of exposure to these factors, constructed through the combination of duration and intensity, increases.

Workers with a worse health status may have been selected out of the workforce earlier or may have been pushed in jobs less exposed to such factors, with the consequence of a shorter duration of exposure to ergonomic factors at work associated with poor health. As health likely influences walking speed, this healthy worker effect may have led to an underestimation of the association between ergonomic factors and walking speed, as well as of their mediating effect. However, such a bias is expected to have affected the results only to a limited extent, as duration of exposure during the whole working life was considered in the study, and not that occurred in more recent years.

The low participation in the survey may have influenced the results, but as the purpose of the study was to examine the relationship between SEP and walking speed, as well as the mediation effect of exposure to biomechanical factors on this relationship, it seems unlikely that the differences observed between participants and non-participants could have influenced the observed results significantly. In fact, to produce a bias in the association between SEP and low walking speed, a differential non-response for both the exposure and the outcome would be necessary. For example, an underestimation of the association between SEP and walking speed would occur if subjects with both low SEP and low walking speed would have higher non-participation. Several studies that assessed the extent of bias in the association between SEP and health outcomes due to non-response in cross-sectional studies did not find significant differences in the observed associations between respondents and non-respondents (57–60).

The study population was composed of subjects in early old age, who may have not yet developed mobility limitations, with the consequence of an underestimation of the SEP gradient in walking speed, compared with studies including older people. Such a choice was determined by constraints imposed by the enrolment criteria of the Constances cohort, which at baseline included only subjects up to 70 years. Future follow-up of this cohort, which will also include walking speed testing, will allow us to assess the mediating effect of lifetime exposure to biomechanical hazards in an older population. On the other hand, the relatively young sample used avoided that too long time elapsed from retirement, limiting the possibility that the observed associations with walking speed have been confounded by unmeasured non-occupational factors that occurred after retirement. Furthermore, very similar SEP proportions mediated by ergonomic factors were estimated restricting the analysis to 60–70 years subjects (data not shown).

Adjustment for height in the reference model (unadjusted for occupational exposures) may have reduced the walking speed gradient by SEP, as this measure is known to be positively correlated with social class (61), but it is an important anthropometric parameter to take it into account, as it may influence the length of the steps and, therefore, walking speed itself.

## Conclusions

In conclusion, the present study confirmed, using baseline data from a large French cohort study, the existence of a direct socioeconomic gradient in walking speed in both sexes but assessed that the contribution of duration of exposure to repetitive work, intense physical work, and lifting/carrying heavy loads to such a gradient was low, with quite similar mediated proportion found using a formal mediation analysis or the risk attenuation method.

## Data Availability Statement

The datasets generated for this study are available on request to the corresponding author.

## Ethics Statement

The studies involving human participants were reviewed and approved by INSERM Comité d'éthique pour la recherche médicale et en santé (Ermes). The patients/participants provided their written informed consent to participate in this study.

## Author Contributions

Ad'E, FR, MG, AD, and AL developed the concept of the manuscript. Ad'E and FR performed the data analyses. Ad'E drafted the manuscript with input from FR, MG, AD, AL, and YR. All authors provided critical feedback, revised the manuscript for intellectual content, and approved the final version.

## Conflict of Interest

The authors declare that the research was conducted in the absence of any commercial or financial relationships that could be construed as a potential conflict of interest.
